# Could vitamin D supplementation play a role against COVID-19?

**DOI:** 10.3389/fimmu.2022.967215

**Published:** 2022-09-12

**Authors:** Bi Li, Shuangshuang Yang, Ning Hou

**Affiliations:** ^1^ Department of Pharmacy, Medical Supplies Center of Chinese PLA General Hospital, Beijing, China; ^2^ Department of Pharmacy, Shandong Provincial Hospital Affiliated to Shandong First Medical University, Jinan, China; ^3^ Graduate Department, Shandong First Medical University (Shandong Academy of Medical Sciences), Jinan, China

**Keywords:** SARS-CoV-2, COVID-19, vitamin D, supportive treatment, clinical studies, outcome of disease

## Introduction

Severe acute respiratory syndrome coronavirus 2 (SARS-CoV-2) is the latest coronavirus to be discovered. Corona Virus Disease 2019 (COVID-19) remains a major threat to global public health. Since the outbreak of the pandemic, we urgently need to find drugs that can be effective against COVID-19. Vitamin D deficiency is commonly observed as a common biochemical abnormality among patients with COVID-19 (1). It is important to emphasize that Vitamin D insufficiency, whether caused by infection, calcium loss with age or decreased digestion and absorption capacity and so on, especially in the elderly, is most likely to increase the risk of developing a long-term disease of COVID-19 (2).

Several clinical studies, such as observational studies and randomized controlled trials (RCTs) have shown that low Vitamin D levels are independently associated with poor prognosis in COVID-19 (**Tables 1**, **2**). In the early stages of COVID-19 outbreak, despite the absence of enough evidence-based medicine research data support, several scientific societies have already recommended supplementing older adults with Vitamin D to prevent the onset of COVID-19 (20). Based on this, we intend to study how much Vitamin D is contributing to prevention and mitigation of COVID-19, combined with more typical and updated research literature, and comment on the current status of relevant clinical research. We attach great importance to encouraging continued high-quality, basic research and clinical studies to explore Vitamin D supplementation options to lower the risk of severe disease progression of patients with COVID-19.

**Table 1:** Summary of observational study information.

Study	Design	Participants	Conclusion
Ling et al. (3)	Multicenter retrospective study	986 participants with COVID-19	Treatment with cholecalciferol booster therapy, regardless of baseline serum 25(OH)D levels, appears to be associated with a reduced risk of mortality in acute in-patients admitted with COVID-19.
Diaz-Curiel et al. (4)	Retrospective observational study	1549 patients hospitalized for COVID-19	Vitamin D deficiency in patients with COVID-19 is correlated with an increased risk of hospital admission and the need for critical care, vitamin D levels do not influence the rate of mortality.
Carpagnano et al. (2020) (5)	Retrospective, observational study	42 patients with acute respiratory failure due to COVID-19	High prevalence of hypovitaminosis D was found in COVID-19 patients with acute respiratory failure, and severe Vitamin D deficiency significantly related to higher mortality risk.
Subramanian et al. (6)	Observational study	472 patients with COVID-19	Extremely low ( < 25 nmol/L) and high (>100 nmol/L) levels of Vitamin D may be associated with mortality risks of patients with COVID-19.
Luo et al. (7)	Cross-sectional study	335 COVID-19 patients	Vitamin D deficiency impacts COVID-19 hospitalization and severity in the Chinese population.
Charoenngam et al. (8)	Retrospective chart review study	287 COVID-19 patients aged ≥ 18 year	There was an independent association between Vitamin D sufficiency defined by serum 25(OH)D ≥ 30 ng/mL and decreased risk of mortality from COVID-19 in elderly patients and patients without obesity.
Hernández et al. (9)	Retrospective case–control study	216 COVID-19 patients and 197 control population	The study did not find any relationship between Vitamin D concentrations or Vitamin deficiency and the severity of the disease.
Vanegas-Cedillo (10)	–	551 COVID-19 patients	Low Vitamin D may contribute to a pro-inflammatory and pro-thrombotic state, increasing the risk for adverse COVID-19 outcomes.

**Table 2:** Summary information on randomized controlled trials.

Study	Design	Participants	Intervention	Comparisons	Results	Conclusion
Fernandes et al. (11)	Multicenter, double-blind, placebo-controlled, randomized clinical trial	200 hospitalized patients with moderate to severe COVID-19	Daily 200,000 IU of Vitamin (n= 101)	Placebo (n= 99)	No significant difference except GM-CSF.	The findings do not support the use of a single dose of 200, 000 IU of Vitamin D3 for the improvement of hospitalized patients with moderate to severe COVID-19.
Murai et al. (12)	Multicenter, double-blinded, randomized, placebo-controlled trial	32 hospitalized patients with moderate to severe COVID-19	200,000 IU ofVitamin D3 (n=16)	Placebo (n = 16)	Not significantly different	A dose of 200, 000 IU of Vitamin D3 did not significantly reduce the length of hospital stay of patients with severe 25-hydroxyVitamin D deficiency and COVID-19
Cannata−Andía et al. (13)	Multicentre, international, randomised, open label, clinical trial	543 patients with moderate-severe COVID-19 disease	Oral bolus of 100,000 IU of cholecalciferol at hospital admission (n=274)	No treatment(n=269)	No significant difference	The administration of an oral bolus of 100,000 IU of cholecalciferol at hospital admission did not improve the outcomes of the COVID-19 disease.
Caballero-García et al. (14)	Double-blind trial	Old patients after COVID-19 infection	Daily 2000 IU Vitamin D (n=15)	Placebo (n=15)	Indicators of muscle damage decreased	Vitamin D supplementationmay contribute to improving the health status and quality of life of COVID-19 patients.
Maghbooli et al. (15)	Multicenter, Randomized, Placebo-Controlled, Double-Blinded Clinical Trial	106 hospitalized patients who had a circulating 25(OH)D3 concentration of <30 ng/mL	Daily 25 mg 25 (OH)D3 orally (n = 53)	Placebo (n = 53)	Treatment with oral 25(OH)D3 was associated with a significant increase in the lymphocyte percentage and decrease in the neutrophil-to-lymphocyte ratio in the patients.	The 25(OH)D3 intervention significantly decreased the NLR in patients with COVID-19 that was associated with improved clinical outcomes.
Shaun Sabico et al. (16)	Multi-center randomized clinical trial	69 patients hospitalized for mild to moderate COVID-19 disease	5000 IU oral Vitamin D3 (n = 36) for 2 weeks	1000 IU oral Vitamin D3 for 2 weeks (standard control) (n = 33)	5000 IU Vitamin D supplementation for 2 weeks caused a significant increase in serum 25(OH)D levels (adjusted p = 0.003); a shorter time to recovery (p = 0.039) and ageusia (p = 0.035).	A 5000 IU daily oral vitamin D3 supplementation for 2 weeks reduces the time to recovery for cough and gustatory sensory loss among patients with sub-optimal vitamin D status and mild to moderate COVID-19 symptoms.
Karonova et al., (17)	Randomized interventional trial	91 health care workers	Cholecalciferol at a dose of 50,000 IU/week for 2 weeks, followed by 5000 IU/day for the rest (n = 45) for 3months	Daily cholecalciferol at a dose of 2000 IU/day for 3months (n = 46)	No significant differences were evident in morbidity between the comparable groups	Neither Vitamin D intake nor Vitamin D deficiency/insufficiency were associated with a decrease in SARS-CoV-2 morbidity.
Murai et al. (18)	Multicenter, double-blind, randomized, placebo-controlled trial	240 hospitalized patients with moderate to severe COVID-19	200,000 IU of Vitamin D3 (n = 120)	Placebo (n = 120)	Not significantly different	The findings do not support the use of a high dose of Vitamin D3 for treatment of moderate to severe COVID-19.
Torres et al. (19)	multicenter, single-blind, prospective, randomized clinical trial	Hospitalized patients with COVID-19, oxygen saturation < 94% and 25(OH)D serum levels < 30 ng/ml	10,000 IU/day of cholecalciferol for 14 days(n=41)	2000 IU/day of cholecalciferol for 14 days(n=44)	The participants with vitamin D supplementation of 10,000 IU/day for 14 days showed improved biochemical and haematological parameters in plasma.	Administration of high doses of vitamin D3 during hospitalization for COVID-19 may improve the inflammatory environment and cytotoxic response against pseudotyped SARS-CoV-2 infected cells, shortening the hospital stay and, possibly, improving the prognosis.

## Mechanistic studies of vitamin D in prevention and mitigation of COVID-19

Why is vitamin D supplementation beneficial for COVID-19? The clues to whether Vitamin D has a reason to fight SARS-CoV-2 can be found in a large number of previous studies, although most of the proofs are circumstantial and associative (21).

As the severity of COVID-19 mainly depends on the presence of the so-called “cytokine storm”, the supplementation of Vitamin D could be a relevant therapeutic strategy to manage hyperinflammation in patients with COVID-19 (22). Since active Vitamin D is a crucial immunomodulator, Vitamin D was demonstrated to possess inhibitory effects on pulmonary inflammation by strongly influencing the functions of inflammatory cells, including DCs, monocyte/macrophages, T cells, B cells and the integrity of structural epithelial cells (23). Moreover, as the main activated form of Vitamin D_3_, 1,25(OH)_2_ D_3_ not only inhibits the secretion of interleukin 12 (IL-12), IL-23, tumor necrosis factor alpha (TNF-α) and interferon-γ (IFN-γ), but also increases the anti-inflammatory cytokine IL-10 and the number of T cell inhibitory molecule PD-1 (24). Another research results showing that, patients with COVID-19 were also proved to have a high-level of interleukin 6 (IL-6) (25). In terms of regulating immunity, Vitamin D can reduce the risk of viral infection through many mechanisms such as innate immunity, adaptive immunity and so on (26). The most common forms of vitamin D supplementation are cholecalciferol (Vitamin D_3_) and ergocalciferol (Vitamin D_2_), while 25(OH) D_3_ is the form of vitamin D_3_ present in the body. When infection happened in the body, the immune system would start recruiting T cells and neutrophils to the site of infection, while scanty levels of circulating 25(OH) D_3_ will debilitate these immunological reactions (27). Some studies have proved that Vitamin D_3_ could contribute to physical barrier to prevention of against trespass of bacteria both in lung epithelium (28) and gastrointestinal tract (29).

As an integral part of the renin-angiotensin system (RAS) pathway, angiotensin-converting enzyme 2 (ACE2) is the host receptor for SARS-CoV-2 entry into intestinal and alveolar cells (30). In a study of genomic screens, ACE2 and FURIN were adopted as baits to build genomic-guides human tissues-tailored maps of upstream regulatory elements. Following the study of 332 human genes encoding Covid-19 protein targets, they found that Vitamin D can change 25% of the expression level while interfering with 70% of the functions of 27 kinds of Covid-19 proteins (31). In an investigation of serum cytokines, chemokines and growth factors in Covid-19 patients, after taking Vitamin D during hospitalization (200, 000 IU), the patients’ granulocyte-macrophage colony stimulating factor (GM-CSF) levels showed a significant group-by-time interaction effect. In this sense, it is suggested that the therapeutic effect of vitamin D may be a result modulation of adequate innate immune response through decreasing the GM-CSF upregulation (11).

## Clinical studies of vitamin D in prevention and mitigation of COVID-19: Observational studies

Given the wide spread worldwide prevalence of COVID-19 in the past, observational studies on Vitamin D supplementation have been conducted in many countries. Overall, these studies mainly used the serum 25(OH)D concentration of COVID-19 patients as an independent factor to analyse its impact on COVID-19-related indicators, including the rate of acute respiratory failure and ICU admission, mortality risk of COVID-19 patients and so on (4–6). In general, the serum concentration of 25-hydroxy Vitamin D (25OHD) < 20 ng/mL (50 nmol/L) is defined as deficiency. For example, in a multicenter retrospective study, 986 COVID-19 patients were divided into Vitamin D levels sufficiency group (>50 nmol/L), insufficiency group (25-50 nmol/L), and deficiency group (<25 nmol/L). Finally, they found that high-dose Vitamin D supplementation (approximately≥280,000 IU in a time period of up to 7 weeks) significantly reduced the risk of death in hospitalized patients with COVID-19, regardless of the patients’ serum baseline level (3). Some cohort studies and case-control studies mainly focused on the relationship between different Vitamin D levels in patients with mortality, mechanical ventilation, and need for intensive care unit (ICU) care during COVID-19 infection. A cross-sectional study showed that individuals with Vitamin D deficiency were more common among patients with COVID-19 infection and severe illness (7). When Vitamin D sufficiency was defined by serum 25(OH)D ≥30 ng/mL, the risk of mortality from COVID-19 in elderly patients and patients without obesity were decreased (8). At the same time, in correlation studies, Vitamin D deficiency not only was associated with COVID-19 prognostic outcomes, but also increased death risk as well as the need for ICU care of infected individuals. However, for the patient dependent on mechanical ventilation, the deficiency had little effect on the risk of treatment (9). Furthermore, in a prognostic correlation study, Vitamin D deficiency (≤12 ng/ml or <30 nmol/L) may contribute to a pro-inflammatory and pro-thrombotic state, increasing the risk for adverse COVID-19 outcomes (10).

As shown in **Table 1**, we still believe that elevated Vitamin D levels are beneficial for the treatment of COVID-19. But it cannot be ignored that the results of cohort studies generally suffer from insufficient follow-up time and follow-up completeness and case-control studies cannot control the influencing factors well. Therefore, correlation in observational studies cannot be equated with causality in actual clinical trials.

## Clinical studies of vitamin D in prevention and mitigation of COVID-19: RCTs

Randomized controlled trials could avoid selection bias, and enhance component comparability through adequate random allocation. Although the included patients were all infected with SARS-CoV-2, there are differences in criteria and methods of patient enrolment. As shown in **Table 2**, in the existing RCTs, patients were randomly assigned to different doses of Vitamin D to test the efficacy of different Vitamin D levels. High-dose supplementation regimens include 200,000 IU of Vitamin D3 and 400,000 IU of Cholecalciferol (11, 12). Limited by different medical conditions, the scale of patients enrolled in different RCTs varies greatly. In some RCTs, Vitamin D supplement was observed as a trend of clinical benefit, but insufficient enrolment of patients was considered as a reason for the lack of conclusion with statistically significant difference. This problem may exist in clinical trials of different scales, whether RCT enrolled as many as 548 COVID-19 patients (13) or only 32 hospitalized patients (18).

Some RCTs validate the clinical safety of high-dose Vitamin D supplementation, and benefit was also observed in many COVID-19 disease outcomes including length of hospital stay, ICU occupancy rate, mortality, improvement of oxygen saturation and prognosis after recovery from SARS-CoV-2 infection (indicators of muscle damage decreased in the elders) (14–16) and protection of health care workers (17). Although some research results have not been significantly different (13, 18; 17; 12), there are still conclusions support Vitamin D as an effective supportive treatment (32). The administration of high doses of Vitamin D_3_ was observed to improve the inflammatory environment and cytotoxic response against pseudotyped SARS-CoV-2 infected cells. At the same time, it also shortens hospital stay and improves the prognosis possibly of COVID-19 patients (19).

Obviously, due to the characteristics of RCT experiments, strict patient inclusion and exclusion criteria lead to limitations in the representativeness bias and external validity (generalizability) of the research results. For example, improper selection of control measures, or exposure of subjects to certain harmful risk factors, will violate the principles of medical ethics. Obviously, some studies haven’t set placebo control groups especially among the older patients with COVID-19. It should be pointed out that the consideration that the elderly are at risk for Vitamin D deficiency is necessary during RCT design.

## Brief introduction of comorbidity, racial disparities and progression of COVID-19 infection under vitamin D deficiency

The incidence of co-infections could be up to 94.2% in COVID-19 cases (33). Both the studies of Vitamin D on the mechanism of action and observational studies had offered evidences that Vitamin D could diminish the risk of acute respiratory tract infections and COVID-19 (34) *via* disrupting the survival and replication of viruses, reducing risk of inflammatory cytokine production, increasing ACE2 concentrations, and maintaining endothelial integrity. Besides, the co-morbid disorders (e.g., diabetes, hypertension, obesity, cancer and HIV) of COVID-19 patients may affect the severity and progression of the SARS-CoV-2 infection (35–39). Comorbidities such as diabetes, obesity, hypertension and so on may also be connected with Vitamin D deficiency (40), while obesity and diabetes are key to possibly promoting exacerbation of COVID-19 infection (41).

Available literature suggests that oral supplementation with vitamin D and GSH precursor (L-cysteine) to increase circulating levels of 25(OH)D may help prevent or reduce the adverse effects of COVID-19 infection, with potential benefits for the African Americans (AA) population (42). Vitamin D supplementation combined with L-cysteine (a GSH precursor) or N-acetylcysteine maybe more effective than supplementation with Vitamin D alone which could prevent cellular damage caused by cytokine storm in SARS-CoV2-infected individuals with comorbid systemic inflammation such as diabetes, obesity and hypertension (43, 44).

There are also racial disparities and inequities associated with Vitamin D insufficiency, and this could exert an effect on COVID- 19 infection (45). AA are more likely to have inadequate or deficient levels of 25(OH)D due to their increased skin pigmentation compared to other races, reducing the skin’s ability to produce vitamin D from sun exposure (43).

## Conclusion

There has been a certain scale of clinical trial studies on the benefits of Vitamin D supplementation in the prevention and mitigation of COVID-19, covering a variety of research types such as cohort studies, case-control studies, and randomized controlled trials. We can believe that more rigorous and effective clinical research data will continue to confirm the exact effect of Vitamin D supplementation for COVID-19. The clinical application of Vitamin D supplementation will play an important role in the prevention and mitigation of COVID-19 disease. Most of the research results proved that Vitamin D supplementation would bring clinical benefits to COVID-19 patients of prevention and treatment (hospital length, mortality, improvement of blood oxygen saturation, prognosis recovery).

It is worth pointing out that the high risk of increased mortality of older patients may due to their potentially severe acute and chronic diseases but not the Vitamin D deficiency. The evidence from cohort studies for clinical decision-making is still somewhat insufficient, but the randomization method in the design of RCTs prevents selection bias, and has good comparability between groups (randomization, external environment), and its measurement and analysis results are more realistic and reliable. Compared with observational studies, positive conclusions with statistically significant differences are still scarce in RCTs of Vitamin D supplementation against COVID-19. Due to its good clinical evidence value, more rigorous and large-sample, multi-center, high-quality RCTs deserve be carry on to provide more stronger evidence support of Vitamin D supplementation against COVID-19.

We have reason to highlight that the screening and enrollment of patients in clinical trials need to take into account of the influence of various factors, and the correction of co-morbid illnesses of enllored patients clinical trials may be necessary.

## Author contributions

NH proposed the study concept and design. BL drafted and edited the text. SY organized and edited the contents of 2 tables. NH was responsible for review, supervision and revision. All authors have read and agree to the published version of the manuscript. All authors contributed to the article and approved the submitted version.

## Funding

This research was supported by the “Key Project of Educational Reform at the First Medical University”, grant number 2021XZ004, Shandong First Medical University (Shandong Academy of Medical Sciences), China.

## Acknowledgments

Thanks to Professor Hou Ning for his help throughout the research process, including proposing research directions and ideas, searching relevant literature, reviewing and supervising the research, etc.

## Conflict of interest

The authors declare that the research was conducted in the absence of any commercial or financial relationships that could be construed as a potential conflict of interest.

## Publisher’s note

All claims expressed in this article are solely those of the authors and do not necessarily represent those of their affiliated organizations, or those of the publisher, the editors and the reviewers. Any product that may be evaluated in this article, or claim that may be made by its manufacturer, is not guaranteed or endorsed by the publisher.
